# Immunoglobulin G4‐related disease accompanying a small intestinal ulcer: A case

**DOI:** 10.1002/deo2.76

**Published:** 2021-11-24

**Authors:** Yuta Yoshidome, Akinori Mizoguchi, Kazuyuki Narimatsu, Shun Takahashi, Dai Hirata, Shinji Ono, Yusuke Onoyama, Seiya Suzuki, Tomoaki Horiuchi, Nanoka Chiya, Keisuke Ikeyama, Hiroyuki Tahara, Akira Tomioka, Suguru Ito, Rina Tanemoto, Shin Nishii, Kenichi Inaba, Nao Sugihara, Yoshinori Hanawa, Kazuki Horiuchi, Akinori Wada, Yoshihiro Akita, Masaaki Higashiyama, Shunsuke Komoto, Kengo Tomita, Shinya Yoshimatsu, Susumu Matsukuma, Ryota Hokari

**Affiliations:** ^1^ Department of Gastroenterology National Defense Medical College Saitama Japan; ^2^ Department of Pathology and Laboratory Medicine National Defense Medical College Hospital Saitama Japan

**Keywords:** endoscopic finding, gastrointestinal ulcer, hypertrophic pachymeningitis, IgG4 related disease, small bowel ulcer

## Abstract

Immunoglobulin (Ig)G4‐related disease (IgG4‐RD) is a systemic condition associated with fibroinflammatory lesions and is characterized by elevated serum IgG4 levels and IgG4‐positive cell infiltration into the affected tissues. It has been reported that IgG4‐RD affects a variety of organs but uncommonly affects the gastrointestinal tract. In particular, there are few cases of lesions in the small intestine, except for sclerosing mesenteritis, which were mostly diagnosed from surgical specimens. Herein, we describe the case of a 70‐year‐old man who initially presented with abdominal pain, headache, later cognitive decline, and gait disturbance caused by IgG4‐RD. Colonoscopy revealed irregular ulcers in the terminal ileum, and computed tomography of the head showed hypertrophic pachymeningitis. Numerous IgG4‐positive cells were detected in the ileal and dural biopsies. We diagnosed the patient with IgG4‐RD and started steroid pulse therapy. After initiation of treatment, the symptoms quickly improved. The patient was discharged from the hospital after starting oral prednisolone treatment (30 mg). The dosage was gradually reduced to 10 mg. A follow‐up colonoscopy revealed scarring of the ileal ulcers. This case may provide valuable information regarding the endoscopic findings of small intestinal lesions in IgG4‐RD.

## INTRODUCTION

IgG4‐related disease (IgG4‐RD), a systemic condition associated with fibroinflammatory lesions, is characterized by increased serum IgG4 levels and infiltration of IgG4‐positive cells into the affected tissues.^1^ IgG4‐positive cell infiltration has been reported to be common in the pancreas, salivary glands, hepatobiliary tract, orbit, lymph nodes, and retroperitoneum, but rare in the gastrointestinal tract.^2^ Previous reports have shown that IgG4‐RD has a wide range of clinical manifestations, including nodules, ulceration, polyps, wall thickening, submucosal tumors, gastrointestinal obstruction due to extramural inflammation, vasculitis, and fistula in the esophagus, stomach, and colon.^3^, ^4^, ^5^ However, there have been few case reports of small intestinal lesions, most of which were secondary changes due to sclerosing mesenteritis diagnosed after surgery. To the best of our knowledge, there has been only one report of small intestinal IgG4‐RD with a primary lesion in the small intestinal mucosa based on endoscopic images.^6^

In this report, we describe a case of IgG4‐related intestinal lesions and endoscopic findings before and after steroid therapy.

## CASE REPORT

**FIGURE 1:**
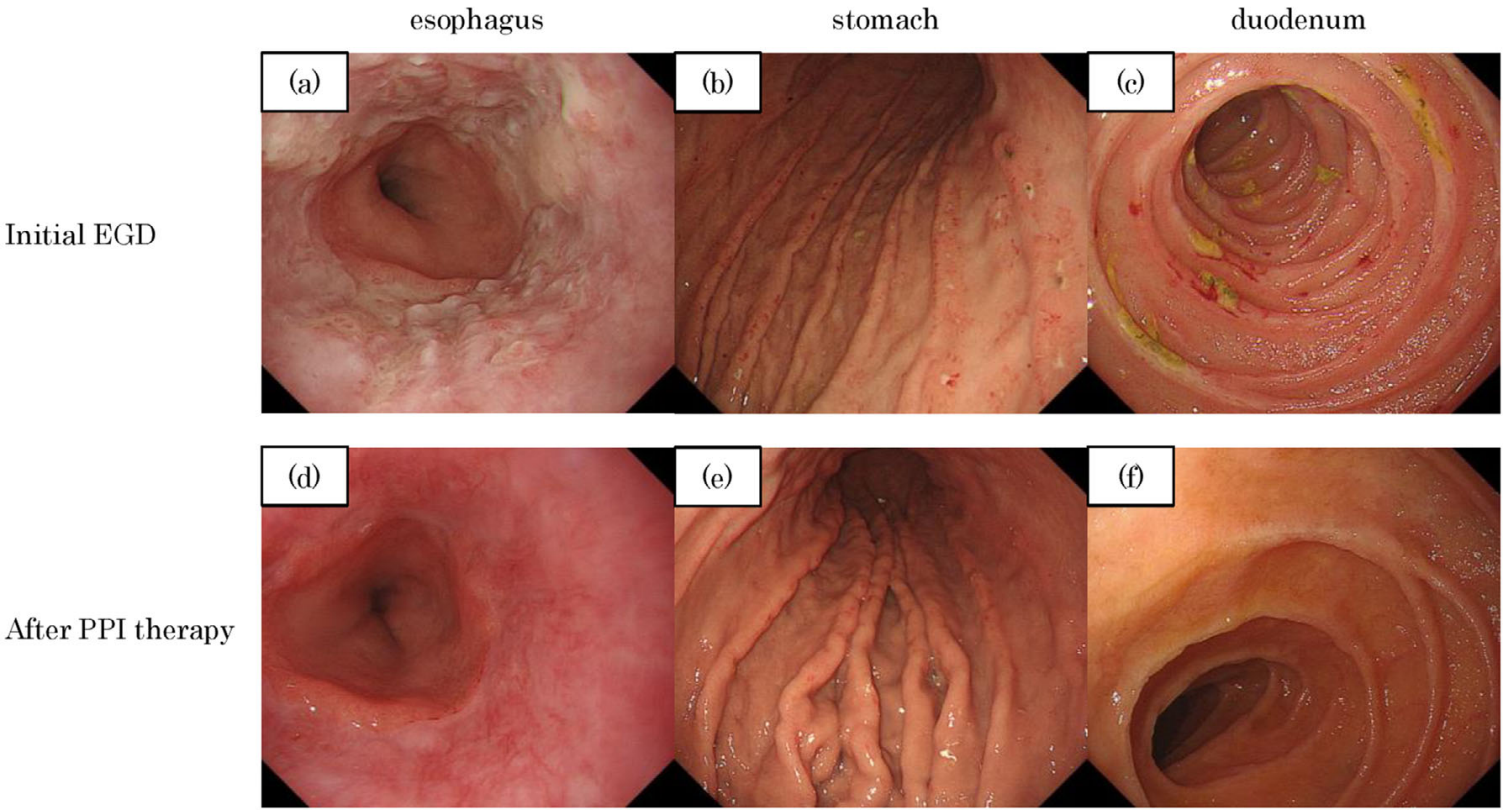
(a–c) Initial esophagogastroduodenoscopy (EGD). (a) Atypical erosions and ulcers of reflux esophagitis were seen in the inferior esophagus. No Immunoglobulin G4 (IgG4)‐positive cells were detected in the biopsy. (b,c) Shallow ulcers and erosions were observed throughout the stomach and from the duodenal bulb to the third portion. No IgG4‐positive cells were detected in the biopsy. (d–f) Follow‐up EGD 1 month after proton pump inhibitor (PPI) treatment. The ulcers and erosions healed and thus were suspected to be caused by non‐steroidal anti‐inflammatory drugs (NSAIDs), not IgG4‐related disease

**FIGURE 2:**
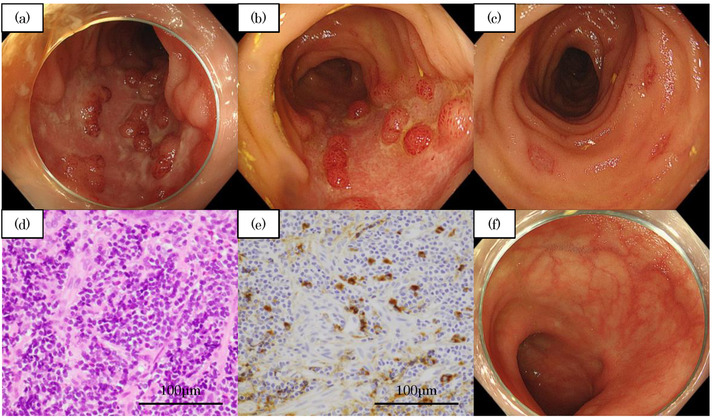
(a) Initial colonoscopy (CS). Extensive map‐like shallow ulcers with no longitudinal trend and well‐defined margins were observed. There was an island of residual mucosa at the fundus of the ulcers. (b,c) Follow‐up CS after 1 month shows the intestinal ulcers and erosions remained unchanged despite healing of the upper gastrointestinal lesion. (d) Hematoxylin Eosin staining of ileal ulcer. It showed moderate infiltration of lymphocytes and no evidence of malignancy or cytomegalovirus infection. (e) IgG4 immunostaining. More than 20 IgG4‐positive cells per high power field (HPF) were detected in the biopsies from ileal lesions. (f) Follow‐up CS during maintenance therapy with 10 mg of prednisolone 2 months after initiation of therapy. The ulcers at the terminal ileum were scarred. No IgG4‐positive cells were detected in the biopsy at this time

**FIGURE 3:**
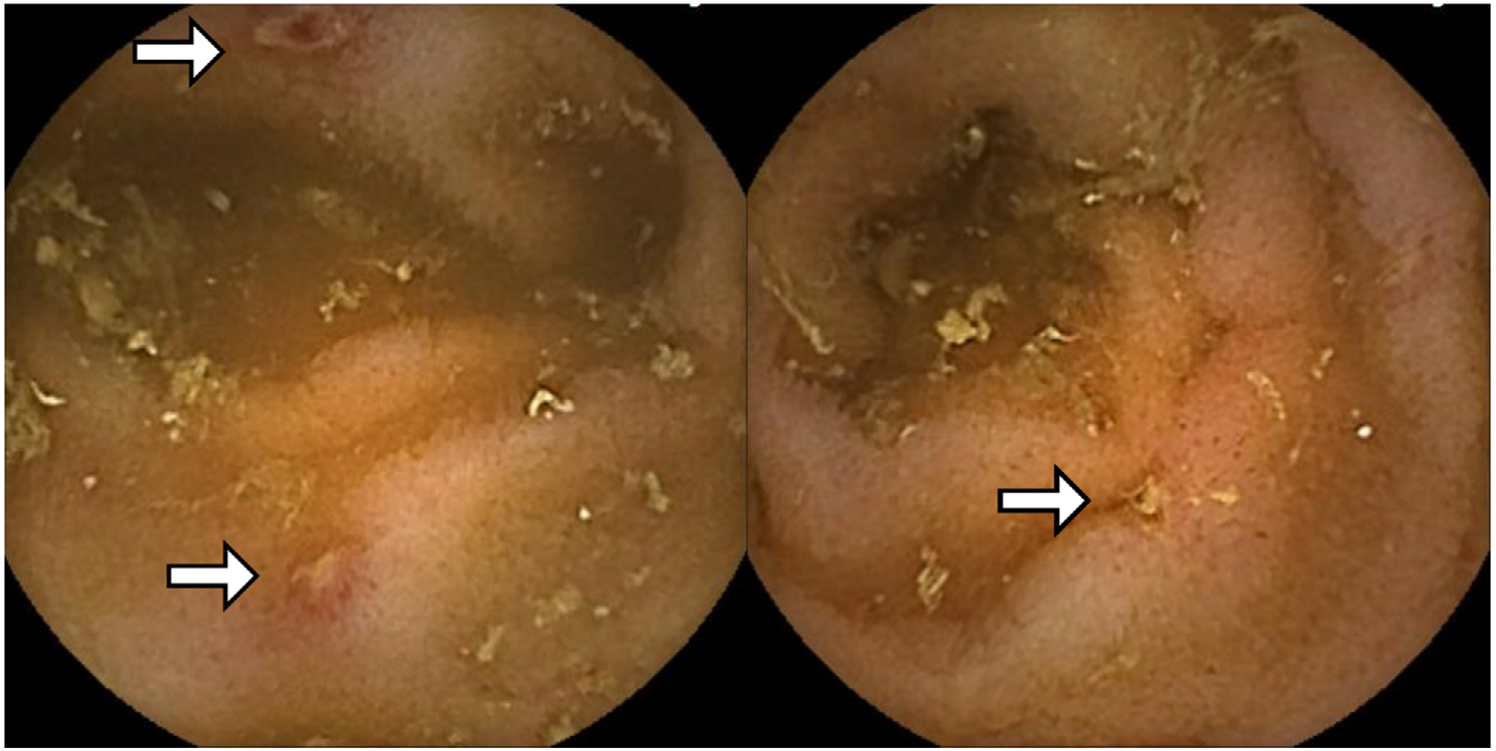
Capsule endoscopy showed scattered aphtha‐like erosions in the ileum, as indicated by arrows

A 70‐year‐old man suffering from abdominal pain and headache visited his family doctor and was prescribed non‐steroidal anti‐inflammatory drugs (NSAIDs) and a mucosal protectant for 2 weeks. He was referred to our hospital because of unresolved symptoms and the onset of melena. On arrival, he showed hemodynamic instability with low blood pressure (80/56 mmHg), high pulse rate (100 bpm), and severe anemia (hemoglobin, 6.8 g/dl). Initial esophagogastroduodenoscopy (EGD) and colonoscopy (CS) were performed immediately after the patient was stabilized by a blood transfusion. EGD showed gastric and duodenal ulcers, potentially due to NSAIDs, and atypical esophagitis lesions for reflux esophagitis (Figure 1a–c). CS showed irregular ulcers in the terminal ileum (Figure 2a) and Hematoxylin Eosin staining of these lesions showed moderate infiltration of lymphocytes (Figure 2d). Biopsy from the endoscopically normal mucosa of terminal ileum demonstrated only mild infiltration of lymphocytes. There were no inflammatory findings in the colon endoscopically or pathologically. We did not perform hemostasis because active bleeding or exposed blood vessels were not observed in either endoscopic examination. Biopsy of all the lesions showed no evidence of malignancy or cytomegalovirus infection. The patient's condition slowly improved after the administration of a proton‐pump inhibitor (PPI). Capsule endoscopy was performed to thoroughly examine the small intestine, and multiple small erosions were seen in the ileum (Figure 3). No pathogenic bacteria were detected in stool culture. From differential diagnosis, infectious enteritis was excluded by the results of stool culture and biopsy. The patient's gastrin level before PPI administration was 100 pg/ml (normal < 200 pg/ml). Serum MPO‐ANCA, PR3‐ANCA, and antinuclear antibodies were negative. In addition, chest, abdominal, and pelvic contrast‐enhanced computed tomography did not reveal abnormal findings. Therefore, we excluded collagen disease, ANCA‐related vasculitis, and Zollinger‐Ellison syndrome. One month later, we performed a follow‐up EGD and CS, which showed that the gastric and duodenal ulcers and esophagitis had healed (Figure 1d–f). However, the terminal ileal ulcers and erosions did not improve despite the discontinuation of NSAIDs (Figure 2b,c). Since the general condition improved, we carefully monitored him as an outpatient.

**FIGURE 4:**
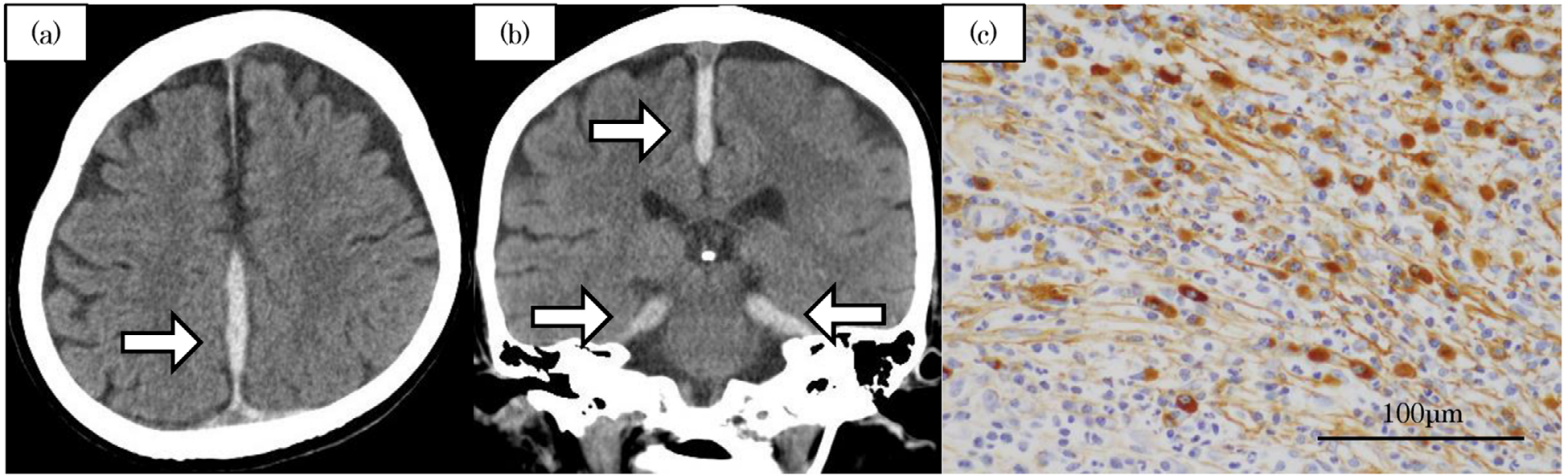
(a,b) Head computed tomography was obtained when cognitive decline, gait disturbance, and worsening of headache were present. The arrows indicated falx cerebri and tentorium cerebelli as high‐density areas. (a) Axial view. (b) Coronal view. (c) IgG4 immunostaining. More than 90 IgG4‐positive cells per HPF were detected in the dural biopsies

One month later, the patient developed cognitive decline (mini‐mental state examination, 21/25), gait disturbance, and worsening of headache. Head computed tomography revealed falx cerebri and tentorium cerebelli as high‐density areas, suggesting hypertrophic pachymeningitis (Figure 4a,b) due to IgG4‐RD. The serum IgG4 level was 393 mg/dl (normal is below 121 mg/dl), the dural biopsy showed more than 90 IgG4‐positive cells per high power field (Figure 4c), and the IgG4‐positive:IgG‐positive ratio was 83.2%. Additional immunostaining of the specimens from the esophagus, stomach, duodenum, and ileum, which were obtained during the previous admission, showed no IgG4‐positive cell infiltration in the esophagus, stomach, and duodenum. However, more than 20 IgG4‐positive cells infiltrated per high power field in the ileal lesion (Figure 2d,e) where the IgG4‐positive:IgG‐positive ratio was over 50%. We diagnosed IgG4‐related disease according to the 2019 American College of Rheumatology and European League against Rheumatism (ACR/EULAR) classification criteria^7^ (the patient's score was 26 and the cut‐off point was 20). After adequate informed consent, the patient underwent steroid pulse therapy, where methylprednisolone was administered at 1000 mg for 2 days and was subsequently tapered to 500 mg for 1 day. After treatment initiation, the symptoms such as cognitive decline, gait disturbance, and headache quickly improved. Subsequent oral treatment with 30 mg/day of prednisolone was started and the patient was followed for 2 weeks before being discharged. The dosage was gradually reduced to 10 mg. A third CS, performed 2 months after the start of steroid treatment, showed improvement and scarring of the ileal ulcers (Figure 2f).

## DISCUSSION

IgG4‐RD was first recognized as a distinct disease in the case of sclerosing pancreatitis reported by Hamano et al. in 2001.^8^ Since then, many cases have been reported, and it has become clear that there is a strong predilection for certain organs, although the disease can affect virtually any organ.^9^ The international classification criteria for IgG4‐RD published in 2019, defined the involvement of the pancreas, salivary glands, bile ducts, orbits, kidneys, lungs, aorta, retroperitoneum, pachymeningitis, and thyroid gland as entry criteria^7^. However, the gastrointestinal tract was not included in these criteria. In addition, infiltration of IgG4‐positive cells was observed in other inflammatory conditions of the gastrointestinal tract, such as inflammatory bowel disease, celiac disease, Rosai‐Dorfman disease, and Cronkhite‐Canada syndrome.^4^ This suggests that an increase in IgG4‐positive cells alone is not specific to IgG4‐related gastrointestinal disease. Therefore, caution should be exercised when diagnosing IgG4‐RD based solely on biopsy results.

In this study, the endoscopic image showed that the terminal ileal lesion was characterized by oval or map‐like shallow ulcers with no longitudinal trend and intact mucosa around the ulcer (Figure 2a,b). Multiple small erosions were observed in a separate part of the ileum (Figures 2c and 3). Differential diagnoses of diseases based on these images include eosinophilic gastroenteritis, malignant lymphoma, and inflammatory bowel disease. However, the ileal ulcers and erosions, in this case, were considered an IgG4‐RD gastrointestinal lesion for the following reasons: (1) The patient was diagnosed with pachymeningitis, which is defined as entry criteria in The 2019 ACR/EULAR classification criteria for IgG4‐RD; (2) Unlike the upper gastrointestinal lesions (Figure 1), the ileal ulcers and erosions did not improve even after NSAIDs administration had been stopped, although the general condition had improved; (3) Biopsy of the ileal lesions showed numerous IgG4‐positive cell infiltrations; (4) The lesion was ameliorated by steroid therapy and appeared scarred on endoscopy with no detectable IgG4‐positive cells after treatment.

There has been only one report on endoscopic images of small intestinal lesions in IgG4‐RD previously by Fujita et al.^6^ The features of the lesions in the report were similar to those in our case, with neither longitudinal nor circular ulcerations. More case reports are needed to determine the characteristics of small intestinal lesions in IgG4‐RD.

In conclusion, we report a case of a small intestinal ulcer accompanied by IgG4‐RD. The lesion responded to steroid therapy and became scarred on improvement. A biopsy is useful for diagnosis, but caution should be exercised because IgG4 cell infiltration may be observed in other diseases. It is difficult to diagnose IgG4‐RD from gastrointestinal lesions alone. Thus, it is necessary to perform a thorough systemic examination and associated symptom check.

## CONFLICT OF INTEREST

The authors declare that they have no conflict of interest.

## FUNDING INFORMATION

None.
